# Genomic signatures of host-range divergence in the generalist *Beauveria bassiana* and the specialist *Beauveria brongniartii*

**DOI:** 10.1007/s00438-026-02506-z

**Published:** 2026-09-11

**Authors:** Alexandra M. Kortsinoglou, Hannah Popp-Embleton, Jürg Enkerli, Hermann Strasser, Vassili N. Kouvelis

**Affiliations:** 1 Section of Genetics and Biotechnology, Department of Biology, National and Kapodistrian University of Athens, 15771 Athens, Greece; 2 Department of Microbiology, Leopold-Franzens University Innsbruck, Technikerstraße 25, 6020 Innsbruck, Austria; 3 Molecular Ecology, Agroscope, Zurich, Switzerland

**Keywords:** *Beauveria*, Entomopathogenic fungi, Comparative genomics, Effectors, Biosynthetic gene clusters (BGCs), Carbohydrate active enzymes (CAZymes), Host specificity

## Abstract

Entomopathogenic fungi of the genus *Beauveria* are widely used biological control agents that infect diverse insect hosts and can also associate with plants as rhizosphere colonizers and endophytes. Within this genus, *Beauveria bassiana* is a cosmopolitan generalist, whereas *Beauveria brongniartii* exhibits a narrower host range, primarily targeting soil-dwelling coleopteran larvae with limited evidence of plant colonization. To explore genomic differentiation associated with this ecological divergence, the commercially exploited *B. brongniartii* strain BIPESCO2 and *B. bassiana* ATHUM 4946 were sequenced using Oxford Nanopore technology, followed by comparative genomic analyses across multiple strains. Orthology identified species-specific gene families, although overall genome architecture and core gene content were highly conserved. The CAZyme repertoires were nearly identical, indicating retention of a versatile enzymatic toolkit supporting plant association, saprotrophy, and insect pathogenicity. In contrast, biosynthetic gene clusters displayed substantial variation, including structural remodeling of *Beauveria*-specific virulence-associated clusters and expansion of type I polyketide synthase clusters in *B. brongniartii*. Effector prediction revealed a conserved core of largely uncharacterized proteins alongside species-specific orthogroups enriched in adhesion-, immunity-, and cuticle-interaction domains. Together, these findings indicate that host-range divergence in *Beauveria* is associated with compartmentalized genomic differentiation, particularly in secondary metabolism and a limited subset of lineage-specific virulence factors, rather than in the conserved core infection machinery.

**Supplementary Information:**

The online version contains supplementary material available at https://doi.org/10.1007/s00438-026-02506-z.

## Introduction

Entomopathogenic fungi (EPF) are soilborne pathogens of a wide range of insects and agricultural pests and are effectively used as biocontrol agents (BCAs) in integrated pest management strategies (Faria and Wraight 2007; Butt et al. 2016; Sharma and Sharma 2021). Among EPF, the genus *Beauveria* (Bals.) Vuill. (Hypocreales: Cordycipitaceae) encompasses both generalist and specialist species, which differ in host range and ecological adaptability. In addition to their entomopathogenic lifestyle, several *Beauveria* strains are also associated with plants as saprotrophs, endophytes, or epiphytes, contributing to plant growth promotion and protection (Vega et al. 2008; Rehner et al. 2011; Jaber and Enkerli 2017).

*Beauveria bassiana* is a cosmopolitan generalist with a global distribution and is among the most extensively studied and widely applied EPF (Feng et al. 1994). It occurs across diverse climatic regions, from Arctic to tropical regions (Ghikas et al. 2010) and infects numerous economically important insect pests across multiple orders, such as thrips, whiteflies, spider mites, and aphids (Boomsma et al. 2014; Ayilara et al. 2023). Beyond its entomopathogenic activity, many *B. bassiana* strains have been isolated as endophytes or epiphytes in plant tissues of a variety of crops, where they have been associated with plant growth promotion and enhanced resistance to pests and diseases (Posada and Vega 2005; Klieber and Reineke 2016; Jaber and Ownley 2018).

In contrast, *Beauveria brongniartii* is generally regarded as a specialist species with a narrower host range, being primarily associated with coleopteran hosts (Piatti et al. 1998; Imoulan et al. 2019). It is considered the principal fungal pathogen of the European cockchafer *Melolontha melolontha* L. (Coleoptera: Scarabaeidae) (Keller et al. 1999; Clifton et al. 2023), a widespread pest in central Europe causing significant agricultural damage (Laengle et al. 2005; Wagenhoff et al. 2014; Sukovata et al. 2015). Since its recognition as a pathogen of this insect, *B. brongniartii* has been established as the main antagonist of both larvae and adults (Büchi et al. 1986; Keller et al. 2003; Dolci et al. 2006; Pedrazzini et al. 2025). Recent experimental work further supports its host-focused virulence strategy, demonstrating that infectivity against *Melolontha melolontha* depends on spore type, infection route, and oosporein production (Graf et al. 2025). Nevertheless, it has demonstrated pathogenicity to other insect species, including *Holotrichia serrata*, *Hypopholis sommeri*, *Exomala orientalis*, and *Spodoptera litura* (Choo et al. 2002; Enkerli et al. 2004; Laengle et al. 2005; Hadapad and Zebitz 2006; Goble et al. 2012; Mayerhofer et al. 2015; Vivekanandhan et al. 2025), and it has also been detected in soils where its typical hosts are absent (Keller et al. 2003). While endophytic colonization has been extensively documented in *B. bassiana*, evidence for such interactions in *B. brongniartii* remains limited (Jaber and Enkerli 2017).

Despite differences in ecology and host range, *Beauveria* species share conserved infection strategies common to Hypocrealean EPF. Infection typically involves adhesion to the insect cuticle, germination of conidia, appressorium formation, penetration of the host integument, proliferation within the hemocoel, and secretion of virulence factors that suppress host immune responses and facilitate colonization (Ortiz-Urquiza and Keyhani 2013; Pedrini 2018). These processes are mediated by diverse molecular mechanisms, including the production of hydrolytic enzymes such as chitinases and proteases (Fan et al. 2007; Meyling et al. 2009; Sánchez-Pérez et al. 2014; Wu et al. 2019), as well as secondary metabolites and other effectors that modulate host physiology and immunity (Fan et al. 2013; Wu et al. 2019, 2020).

Comparative genomic studies in entomopathogenic fungi have highlighted the importance of genes associated with host interaction and virulence, including secreted proteins and effectors that mediate host immune suppression and facilitate tissue colonization (Xiao et al. 2012; Rovenich et al. 2014; Staats et al. 2014), carbohydrate-active enzymes (CAZymes), which aid in substrate degradation and nutrient acquisition (Gao et al. 2011; Xiao et al. 2012; Zhao et al. 2013; Fan et al. 2017), and secondary metabolite biosynthetic gene clusters (BGCs) that have multiple roles during infection (Xu et al. 2009; Meng et al. 2021; Pedrini 2022). Omics-based approaches have further advanced our understanding of these pathogenicity determinants, particularly in *B. bassiana* (Harith-Fadzilah et al. 2021). Variation in these functional categories has been linked to differences in virulence, ecological specialization, and host range, suggesting that they represent key determinants of host adaptation.

Genomic resources are abundant for *B. bassiana* (Xiao et al. 2012; Valero-Jiménez et al. 2016a, b; Castro-Vásquez et al. 2022; Lee et al. 2024), whereas available data for *B. brongniartii* remain limited, and comprehensive comparative analyses of virulence-related gene repertoires across strains are still lacking. To address this gap and investigate the genomic basis of host-range divergence within the genus, we generated high-quality genome assemblies for *B. brongniartii* strain BIPESCO2 and *B. bassiana* strain ATHUM4946. The *B. brongniartii* assembly provides an improved genomic reference for the species, as most previously available assemblies are fragmented. For comparative purposes, the newly sequenced *B. bassiana* genome was analysed alongside existing genomic resources.

In this study, we specifically aimed to: (i) generate a high-quality genomic reference for *B. brongniartii*; (ii) perform a genome-wide comparison between a specialist (*B. brongniartii*) and a generalist (*B. bassiana*) across multiple strains; and (iii) assess whether differences in orthogroup composition, carbohydrate-active enzymes, secondary metabolite biosynthetic gene clusters, and effector repertoires are associated with host-range divergence. By integrating these analyses across 20 genomes representing diverse ecological and geographic origins, we sought to identify lineage-specific genetic features that may contribute to the contrasting ecological strategies of the cosmopolitan generalist *B. bassiana* and the more host-restricted *B. brongniartii*.

## Materials and methods

### Fungal species

*B. brongniartii* strain BIPESCO2 was isolated in Austria from *M. melolontha* (Strasser et al. 2003). *B. bassiana* ATHUM4946 was isolated from air samples in Athens, Greece and was acquired by Mycetotheca ATHUM, the Dried Specimen and Culture Collection of Fungi at the National and Kapodistrian University of Athens (NKUA, Athens, Greece). Strains were cultivated in Potato-Dextrose-Agar (PDA) medium for 7 days in the dark, at 25 ± 1 °C. DNA extraction was performed using the HigherPurity Plant DNA Purification Kit (Canvax Biotech, Spain) using 100 mg of fungal material, according to the manufacturer's protocol. DNA quality and quantity were assessed by electrophoresis (0.8% agarose gel), as well as Nanodrop and Qubit measurements.

### Whole genome sequencing, genome assembly and annotation

Nanopore sequencing was performed using the MinION Mk1b (Oxford Nanopore, UK) device. The R10.4.1 (FLO-MIN114) and R9.4.1 (FLO-MIN110) flow cells were used for *B. brongniartii* BIPESCO2 and *B. bassiana* ATHUM4946 strains, respectively. The sequencing libraries were prepared using the ligation sequencing kits SQK-LSK114 (Q20 + chemistry) and SQK-LSK109 (Oxford Nanopore Technologies), following the manufacturer’s protocol. Sequencing was carried out using MinKNOW (v4.2.5) software. Basecalling was performed locally with Guppy Software (v6.4.6) (Oxford Nanopore Technologies), using the super accurate (SUP) model.

Following basecalling of BIPESCO2, the quality of the obtained reads was verified using Nanoplot (De Coster and Rademakers 2023). Based on the results, raw reads were quality filtered using NanoFilt v2.8.0 (De Coster and Rademakers 2023), retaining reads with a minimum average quality score of Q>10 and a minimum length of 1000 bp. Filtered reads were used for *de novo* assembly with Flye (v2.9.5) (Kolmogorov et al. 2019). The respective assembly was further polished using four rounds of polishing with Racon (v1.5.0) (Vaser et al. 2017) and one final round of polishing with Medaka (v2.1.0) (Oxford Nanopore Technologies Ltd). For strain ATHUM 4946, basecalling and adapter removal were performed as described previously. Reads were corrected and trimmed using Canu (v2.0) (Koren et al. 2017), and the final assembly was performed using Flye (Kolmogorov et al. 2019). Genome annotations were conducted using the GenSAS v6.0 pipeline (Humann et al. 2019), unless otherwise indicated. Low-complexity regions and repetitive elements were identified and masked using RepeatModeler (v2.0.1) (Flynn et al. 2020) and RepeatMasker (v4.1.1) (Smit et al. 2013), with the DNA source set to fungi and the speed/sensitivity parameter configured to slow. A masked consensus sequence was generated and utilized for *ab initio* gene prediction. Transcript alignments were performed with the BLAST nucleotide (blastn) tool (v2.12.0) (Camacho et al. 2009) and BLAT tool (v2.5) (Kent 2002), against the NCBI RefSeq Fungi transcript database. Protein alignments were performed using DIAMOND (v2.0.11) (Buchfink et al. 2021) against the NCBI RefSeq fungi protein database. Gene predictions were performed using Augustus (v3.4.0) (Stanke and Morgenstern 2005), along with *ab initio* prediction using GeneMark-ES (v4.48) with default parameters (Ter-Hovhannisyan et al. 2008), reporting genes on both strands, allowing incomplete gene predictions at sequence boundaries. A consensus gene set was generated by integrating ab initio gene predictions with protein and nucleotide alignments using EvidenceModeler (Haas et al. 2008). Gene set completeness was evaluated using BUSCO (Manni et al. 2021), and based on this assessment, the GeneMark-ES predictions were selected as the official gene set (OGS) (Ter-Hovhannisyan et al. 2008). Functional analysis of the OGS was performed using (1) BLAST protein vs. protein (blastp) against Protein Data Set: NCBI refseq fungi (Camacho et al. 2009), (2) DIAMOND Functional version 2.0.11 against Protein Data Set: NCBI refseq fungi (mode: sensitive, Buchfink et al. 2021), and (3) InterProScan version 5.53-87.0 (Jones et al. 2014). Ribosomal RNA (rRNA) genes were identified using RNAmmer (v1.2) (Lagesen et al. 2007), and tRNA genes were annotated using tRNAscan-SE (v2.0.7) (Chan and Lowe 2019).The official protein set was annotated against the nonredundant (NR) protein sequence (Sayers et al. 2022) database, as well as SwissProt and TrEMBL using Blast2GO suite (Götz et al. 2008). In addition, COG and Pfam annotations were obtained using EggNOG-mapper implemented in Galaxy, to assign functional annotations to the predicted proteins (Cantalapiedra et al 2021). Metabolic pathway enrichment analysis was performed using the Clusters of Orthologous Genes (COG) and KEGG database. KEGG annotations were obtained using GhostKOALA (Kanehisa et al. 2016). Gene Ontology (GO) function enrichment analysis predicted the biological function of the protein sequence encoded by the gene (Harris et al. 2004).

EffectorP (v3.0) was used to detect effector proteins (Sperschneider and Dodds 2022). SignaIP (v6.0) was used to find proteins with signal peptides (Petersen et al. 2011) and TMHMM (v2.0) to predict transmembrane helices in proteins (Krogh et al. 2001). Only proteins with a signal peptide and no transmembrane domain prediction, unless they were within the first 35 amino acids, were retained and were used as input for EffectorP. Antismash v.8.0 was used for the detection of putative biosynthetic gene clusters (Blin et al. 2025). Sequence assemblies as well as gene GFF files were used as inputs with default parameters. An additional similarity network analysis was employed to investigate the similarity of BGCs among all strains, using the program BiG-SCAPE (v2.0.0) (Navarro-Muñoz et al. 2020). All reference BGCs found in the MIBiG database were included to identify similarities with known products (Terlouw et al. 2023). Search for CAZymes was performed using dbCAN3 server (Zheng et al. 2023), which performs automatic CAZyme annotation using Diamond-CAZy, HMMER-dbCAN-substrate and HMMER dbCAN tools. Results supported by at least two tools were considered valid and were used for further analyses.

### Assembly metrics and completeness checks

To evaluate assembly quality, a combination of structural, gene content-based, and k-mer-based validation approaches was applied. Assembly statistics were computed using *seqkit* (v2.3.1) (Shen et al. 2016). Filtered reads were aligned to the assembly using Minimap2 (v2.26) (Li 2018), and mapping statistics along with per-base coverage depth were calculated using Samtools (v1.17) (Danecek et al. 2021) to evaluate coverage uniformity and identify regions of low or excessive depth. Assembly structural accuracy was evaluated with Inspector (v1.3) (Chen et al. 2021), which detects structural inconsistencies such as misassemblies, inversions, and haplotype switches based on alignment features. BUSCO v6.0.0 (Manni et al. 2021) was used to assess gene content completeness by quantifying the recovery of conserved single-copy orthologs from a Hypocreales_odb12 fungal lineage dataset. To independently estimate genome size, repeat content, and read error rates, k-mer frequency distributions were generated using Jellyfish (v2.3.0) (Marçais and Kingsford 2011) and modeled using GenomeScope 2.0 (Ranallo-Benavidez et al. 2020). Contigs identified as low-coverage, high-coverage, or otherwise anomalous were compared to the full assembly using BLAST + to assess redundancy or uniqueness.

### Comparative analysis dataset

**Fig. 1:**
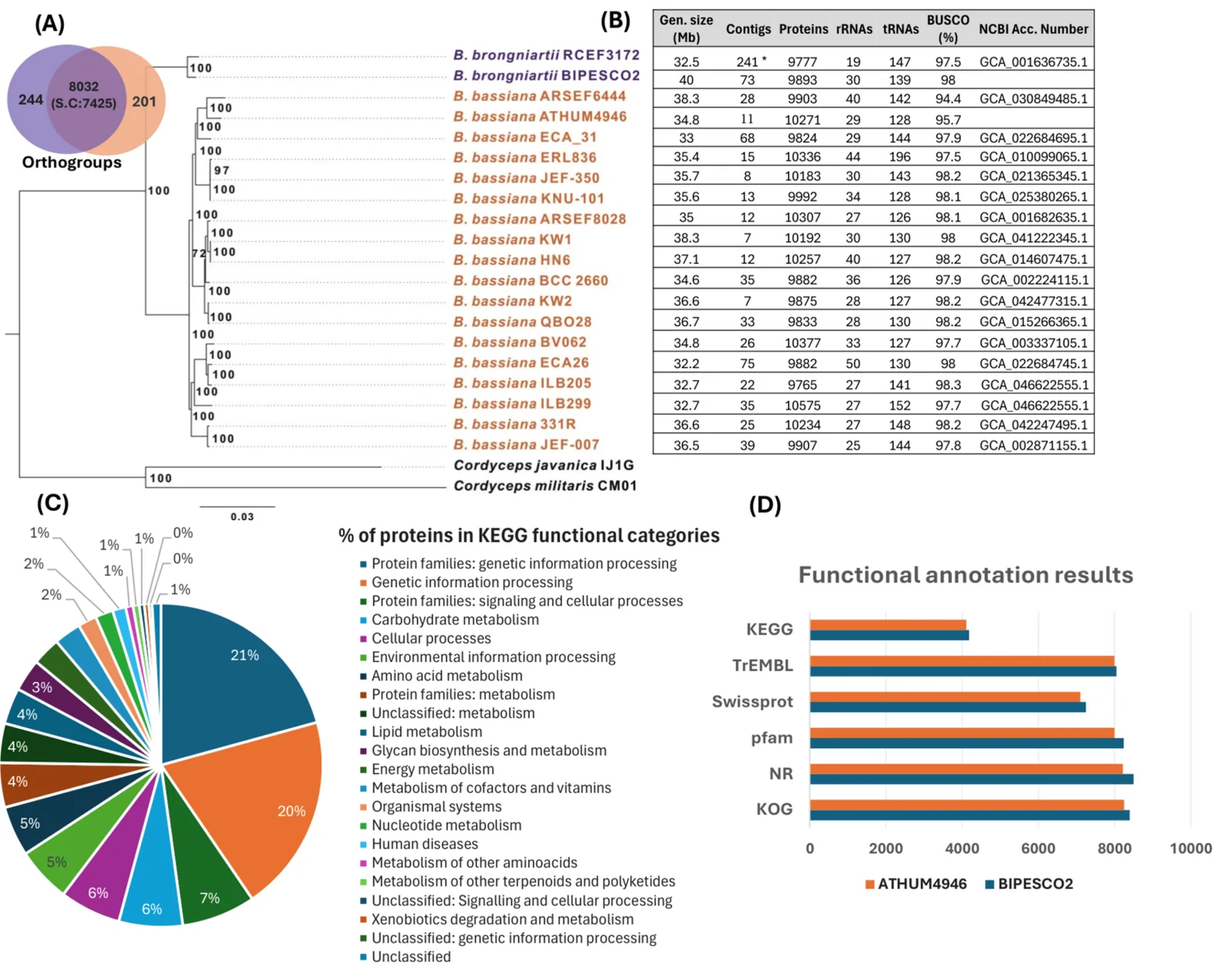
Comparative genomic overview of *Beauveria brongniartii* and *Beauveria bassiana* strains used in this work. **A** Orthogroup clustering of 20 *Beauveria* genomes showing shared and species-specific gene families. A total of 8,032 orthogroups were conserved across all strains (of which 7,425 were single-copy), while 244 were unique to all *B. brongniartii* strains and 201 were unique to all *B. bassiana* strains. The phylogenetic tree was constructed with IQ-TREE using the LG+I+G4 model and a concatenated matrix of single-copy orthologous proteins. **B** Assembly statistics for the newly sequenced genomes (*B. brongniartii* BIPESCO2 and *B. bassiana* ATHUM4946) and 18 publicly available strains, which were re-annotated in this work, including genome size, contig number, predicted protein count, rRNA/tRNA copy number, BUSCO proteome completeness (Hypocreales odb12 dataset), MAT genes, and NCBI accession number. The asterisk (*) indicates that this assembly is at the scaffold level. **C** Distribution of predicted proteins of BIPESCO2 and ATHUM 4946 into KEGG functional categories, illustrating broadly similar metabolic capacities across the two strains, dominated by protein families involved in genetic information processing, signalling and cellular processes, and metabolism. **D** Functional annotation summary for the two newly sequenced genomes against KOG, NR, Pfam, SwissProt, TrEMBL, and KEGG databases.

Comparative analysis was conducted by incorporating publicly available genome assemblies of *B. brongniartii* and *B. bassiana* from the NCBI database, including assemblies generated in previous genomic studies (Xiao et al. 2012; Valero-Jiménez et al. 2016a, b; Lee et al. 2017, 2024; Kim et al. 2016, 2022; Toopaang et al. 2017; Shang et al. 2016; Castro-Vásquez et al. 2022; Liu et al. 2025; Oberti et al. 2024). From a total of 206 available *B. bassiana* assemblies, only 18 with fewer than 100 contigs were retained for downstream analysis to ensure high-quality comparative results. Similarly, one additional *B. brongniartii* genome assembly (with < 900 contigs) was included, resulting in a dataset of 20 strains in total (Fig. 1). To ensure consistency across datasets, all genomes were re-annotated using the same pipeline applied to the newly sequenced *B. brongniartii* *strain* BIPESCO2 and *B. bassiana* strain ATHUM 4946.

### Orthology inference

Orthology inference and phylogenomic reconstruction were performed using OrthoFinder (v2.5.5) (Emms and Kelly 2019) with default parameters, enabling the identification of orthologous groups from the whole proteomes. The construction of a phylogenetic species tree was performed using IQ-TREE 2 (Minh et al. 2020) and was based on single-copy orthologs, as implemented in OrthoFinder. IQ-TREE 2 automatically evaluated alternative models using ModelFinder and identified LG+I+G4 as the best-fitting model for the dataset. Two *Cordyceps* species were used as outgroups.

## Results

### Genome assemblies and annotation

*De novo* assembly of *B. brongniartii* strain BIPESCO2 produced a high-quality genome of 40.13 Mb distributed across 73 contigs (66 > 10 kb). The assembly had strong contiguity, with an N50 of 1.95 Mb and a maximum contig length of 3.69 Mb, values comparable to other reference-grade *Beauveria* genomes. Quality assessment using Inspector confirmed the assembly’s completeness, with 99.96% of reads mapping back and no detected structural errors (expansions, collapses, inversions, or misjoins = 0). The estimated base-level quality value (QV) of 40.6 corresponded to ~ 99.99% consensus accuracy (Table S1). Minor small-scale discrepancies (≈ 84 per Mb, mostly short indels typical of long-read data) were detected but did not affect overall assembly integrity. The mean sequencing depth of 124x ensured comprehensive coverage across the genome. BUSCO assessment using Hypocreales_odb12 database on the official protein set showed 98% complete BUSCOs (Fig. 1). Altogether, these results demonstrated that the *B. brongniartii* BIPESCO2 assembly is highly contiguous, accurate, and nearly complete, providing a reliable genomic reference for downstream comparative analyses. *De novo* assembly of *B. bassiana* strain ATHUM 4946 resulted in a 34.7 Mb genome assembly of 11 contigs, with N50 = 4 Mb and 40x coverage (Table S1). The BUSCO completeness score was 95.7% (Fig. 1), while the respective quality metrics are presented in Table S1. The assemblies have been deposited in the European Nucleotide Archive at EMBL-EBI under accession numbers GCA_978021795 for *B. brongniartii* BIPESCO2 and GCA_981691435 for *B. bassiana* ATHUM4946.

KEGG classification of 10,575 proteins of BIPESCO2 and 10,271 proteins of ATHUM 4946 annotated 4,180 (39.5%) and 4,109 (40.3%) entries, respectively, with most of the proteins of both strains belonging to genetic information processing (41%), signalling and cellular processes (7%), and carbohydrate metabolism (6%). Annotation results against COG, Pfam, and SwissProt resulted in a large proportion of annotated proteins per strain (Fig. 1; Table S2).

### Orthology inference and phylogenetic relationships

Phylogenetic analysis based on single-copy orthologue proteins of all *Beauveria* strains, using two *Cordyceps* spp. as outgroups, separated the *Beauveria* strains into two distinct clades, which is consistent with their current taxonomic species classification (Shen et al. 2020; Kumar et al. 2022). All *B. bassiana* strains formed a tightly clustered group, reflecting their high genomic similarity and conserved gene content (Fig. 1). The two *B. brongniartii* strains formed a monophyletic clade with slightly longer branch lengths and deeper divergence relative to *B. bassiana* strains, consistent with their distinct evolutionary trajectory. The mating type locus of *B. brongniartii* BIPESCO2 was confirmed to contain the MAT1-1-1 gene, using the RCEF 3172 gene (BBO06976) as a reference. No additional MAT genes were identified, in agreement with previous findings for this species (Pedrazzini et al. 2024).

**Fig. 2:**
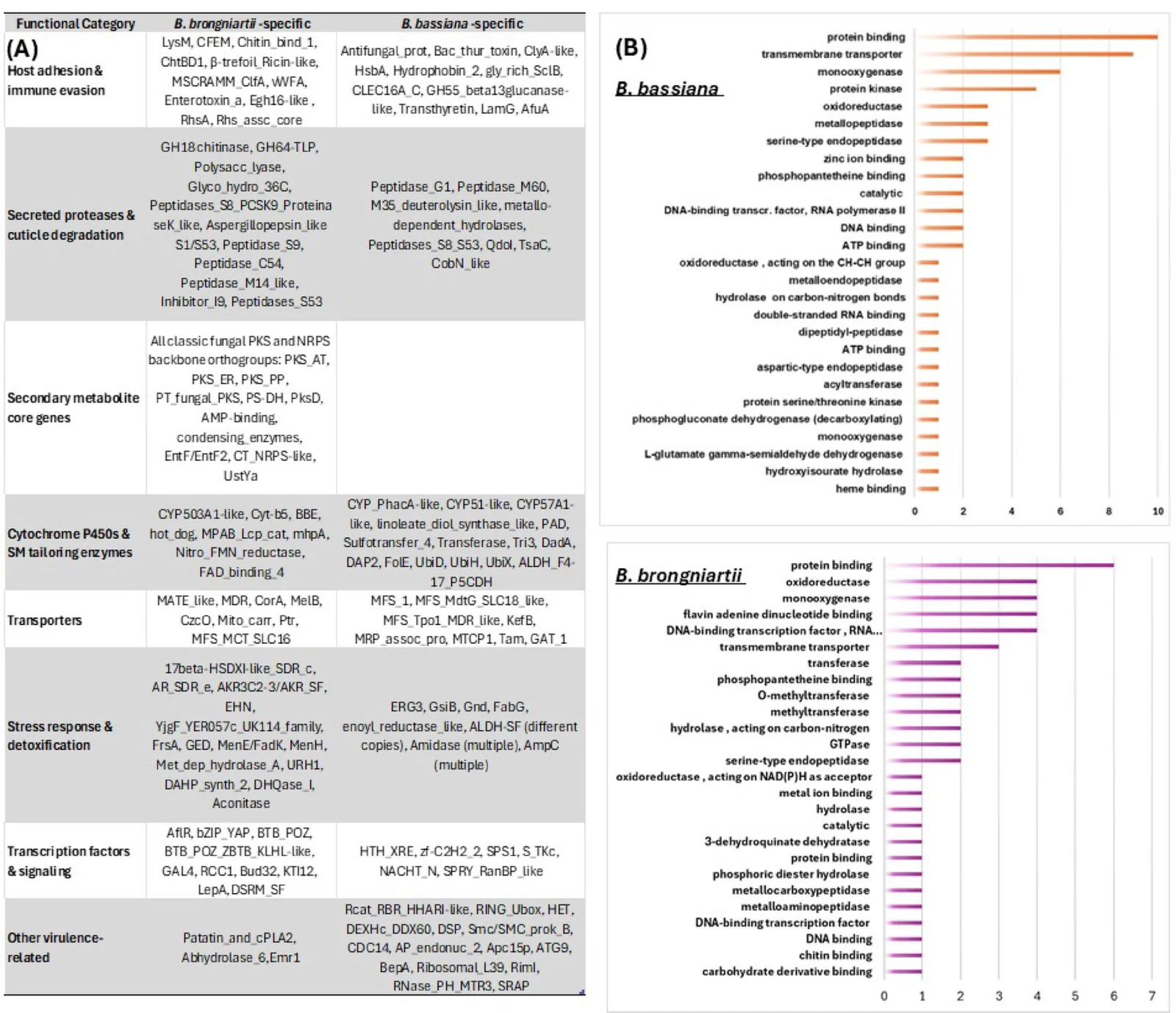
Species-specific orthogroups of *B. brongniartii* and *B. bassiana* and functional classification based on conserved protein domains. **A** Major functional categories of protein domains in species-specific orthogroups. Species-specific orthogroups were present in all strains of one species and absent from all strains of the other. **B** InterProScan GO enrichment (molecular function) annotations of species-specific proteins. *B. bassiana* shows enrichment in protein binding, transporter activity, monooxygenases, and proteases, while *B. brongniartii* is enriched in oxidoreductases, FAD-dependent enzymes, transcription factors, and carbohydrate-binding functions. Large fractions of proteins in both species lacked GO terms, reflecting divergence of lineage-specific genes.

Orthology analysis using only *Beauveria* strains assigned 200,848 genes (99.4% of total) to 11,642 orthogroups. Half of all genes were in orthogroups with 20 or more genes (G50 was 20) and were contained in the largest 4834 orthogroups (O50 was 4,834). A total of 8,032 orthogroups contained representatives from every strain, indicating a large, shared core genome. Notably, 7,425 of these consisted entirely of single-copy genes. Using a strict species specificity criterion (present in all strains of one species and absent from all strains of the other), we identified 201 *B. bassiana*-specific and 244 *B. brongniartii*-specific orthogroups (Fig. 1). These included both single-copy and multi-copy expanded families, with specificity arising either from the complete absence in the opposite species or from sequence divergence sufficient to place them into distinct orthogroups. All species-specific proteins belonged to broad functional categories implicated in host surface interaction, proteolysis, transport, secondary metabolism, regulation, and stress response (Fig. 2; Table S3), but conserved domains varied between the two species (Fig. 2). To further characterize the functional landscape of species-specific orthogroups, InterPro2GO molecular function annotations were examined. *B. bassiana*-specific proteins were enriched in broad metabolic and stress-tolerance functions, including protein binding, transmembrane transporter activity, monooxygenase/oxidoreductase activity, protein kinase activity, and several serine- and metallopeptidases (Fig. 2; Table S3). These GO categories reflect the heterogeneous mix of detoxification enzymes, transporters, and signalling proteins identified in the domain-level orthogroup analysis. In contrast, *B. brongniartii-*specific proteins showed a narrower, but more specialised GO profile enriched for FAD-dependent oxidoreductases, monooxygenases, O-methyltransferases, RNA polymerase II-specific transcription factors, GTPases, chitin- or carbohydrate-binding activities and polyketide synthase (PKS)-associated cluster genes. Both species contained a large proportion of proteins lacking GO terms, reflecting the high divergence typical of species-restricted gene families (Fig. 2; Table S3).

In *B. bassiana*, species-specific orthogroups included multiple adhesion-related proteins (Fasciclin, a triple Hydrophobin_2-domain protein, HsbA, LamG, CLEC16A_C, SclB-like), along with a heterogeneous mix of hydrolases and proteases (M35 deuterolysins, M60 metalloproteases, specialized to break down peritrophic insect gut membrane, abhydrolases, amidases) (Fig. 2; Table S3). In addition, orthogroups with expanded transporter families of multidrug-efflux-associated MFS subfamilies (MFS_MdtG, MFS_Tpo1/MDR-like) and detoxification-related systems (MRP-associated proteins, KefB, AmpC β-lactamase-like enzymes) were found. A notable *B. bassiana* single-copy orthologue encoded a Delta-endotoxin CytB-like protein, homologous to the pore-forming toxins of *Bacillus thuringiensis*, also detected in several other fungal species, such as *Apiospora kogelbergensis* and *Gloeophyllum trabeum* (Fig. 2; Table S3).

In *B. brongniartii*, species-specific orthogroups had several adhesion and surface host-interaction domains, such as chitin- and carbohydrate-binding modules (LysM, CFEM, a GH18 protein with a CBM1 domain), an Egh16-like domain that is an appressorium-specific virulence factor, a peptidase _s8_s53 with a kuma domain, Tryp_SPc serine proteases, and other cuticle-associated hydrolases, which formed separate orthogroups, indicating interspecies sequence divergence (Fig. 2; Table S3). Transporter profiles also differed: *B. brongniartii* encoded a wider range of nutrient, ion, and efflux transport systems (AA_permease_2, MFS_MCT_SLC16 (monocarboxylate permease), MDR, MATE-like, CorA (truncated), MelB, CzcO redox-regulated heavy-metal efflux, mitochondrial carrier proteins as well as regulatory proteins such as the AflR (aflatoxin/sterigmatocystin pathway activator), bZIP_YAP (oxidative-stress response), GAL4-like Zn₂Cys₆ factors, BTB/POZ-domain proteins, RCC1 (Ran-GEF), the KEOPS/EKC complex components Bud32 and KTI12 (tRNA modification and telomere homeostasis), LepA (ribosomal back-translocase). Additional species-specific proteins included: (i) Emr1 (Pfam17237), an ERMES-associated regulatory component of the Endoplasmatic Reticulum (ER)-mitochondria encounter structure, involved in phospholipid exchange and mitochondrial-ER stress responses in yeasts (Kakimoto-Takeda et al. 2022), conserved in *Metarhizium*, *Purpureocillium*, and *Emericellopsis*; (ii) a double-stranded RNA-binding motif (DSRM_SF, cd00048) protein, with homologs in *Cordyceps* and plant-associated fungi; (iii) an Rhs-associated core module protein (PF05593), a polymorphic, multidomain toxin originally described in bacteria (especially Gram-negative) that was only found in *Akanthomyces muscarius* and several other bacteria (iv) an Enterotoxin_a-like protein also present in *Cordyceps militaris* and *Metarhizium* sp., and (v) a PKS, that is only found in *Cordyceps militaris* CM01 strain with 100% coverage and 87% similarity, while other fungi such as *Colletotrichum* sp., *Podospora* sp. and *Sodiomyces alcalophilus* showed lower % similarity (58%) than the *C. militaris* strain. These proteins collectively represent a set of lineage-specific loci absent from all *B. bassiana* genomes (Fig. 2; Table S3).

Moreover, analysis of the number of selected proteins known to be associated with fungus- host interactions, based on Pfam annotation, showed highly similar repertoires of proteins involved in host penetration, adhesion, and detoxification, indicating a conserved entomopathogenic core (Table 1).

**Table 1:** Counts of protein domains associated with fungus-host interactions. Annotations were based on Pfam database.

Broad functional category	Domains	PFam accession numbers	*B. brongniartii*		*B. bassiana*																	
			BIPESCO2	RCEF3172	ARSEF 8028	ECA_26	ECA_31	ERL836	HN6	ILB205	ILB299	JEF-007	JEF-350	KNU-101	KW1	KW2	ATHUM 4946	QB028	331R	ARSEF 6444	BCC2660	BV062
Proteases	Subtilisin-like serine protease (family S8)	PF00082	42	42	40	41	43	40	41	41	40	38	46	47	41	41	46	41	39	44	40	43
	Subtilisin-like serine protease propeptide (pro-kumamolisin activator domain)	PF09286	17	18	15	15	16	15	15	15	15	15	15	15	15	15	15	15	15	16	15	15
	Subtilisin-like serine protease propeptide	PF05922	1	1	1	1	1	1	1	1	1	0	1	1	1	1	1	1	1	1	1	1
	Trypsin-like serine protease	PF00089	15	13	16	15	21	26	19	16	15	19	26	21	19	24	26	24	18	19	16	19
	Trypsin-like serine protease propeptide	PF03372	1	1	1	1	1	1	1	1	1	1	1	1	1	1	1	1	1	1	1	1
	Metalloprotease, family M28	PF02156	14	12	13	13	12	12	13	13	15	13	12	12	13	12	13	12	13	13	15	14
	Fungalysin metalloprotease (family M36)	PF02128	0	0	0	0	0	0	0	0	0	0	0	0	0	0	0	0	0	0	0	0
	Metalloprotease, family M43	PF05543	8	9	7	8	7	9	6	7	6	6	8	9	6	8	9	8	6	10	7	7
	Papain-like cysteine protease (family C1)	PF00112	10	11	10	10	10	9	10	11	11	11	9	9	10	10	10	10	12	10	10	9
	Legumain-like cysteine protease (family C13)	PF01650	1	1	1	1	1	1	1	1	1	1	1	1	1	1	1	1	1	1	1	1
	Caspase-like cysteine protease (family C14)	PF00656	3	4	4	4	4	3	4	4	4	5	3	3	4	4	4	4	6	4	4	4
	Aspartic protease	PF00026	1	1	1	1	1	1	1	1	1	1	1	1	1	1	1	1	1	1	1	1
Surface / adhesion	Hydrophobin	PF01185	5	5	5	5	5	5	5	5	5	5	5	5	5	4	5	5	5	5	5	5
	Hydrophobin_2	PF06395	3	3	3	3	3	3	3	3	3	3	3	3	3	2	3	3	3	3	3	3
	CFEM	PF05730	17	17	15	15	15	16	17	15	15	15	16	15	17	16	15	16	15	17	15	16
	Cerato-platanin	PF07249	2	2	2	2	2	2	2	2	2	2	2	2	2	2	2	2	2	2	2	2
	Fasciclin-like adhesion protein	PF02469	3	3	4	4	4	4	4	4	4	4	4	4	4	4	4	4	4	4	4	7
	Flocculin type III repeat	PF00643	5	5	4	4	4	6	5	4	4	5	6	7	5	5	4	5	4	6	4	5
	WSC cell wall stress–responsive sensor domain	PF01822	15	16	15	15	14	15	15	15	15	16	15	15	15	16	15	16	17	15	15	17
	Hyphal cell wall protein regulatory domain	PF11746	1	1	1	1	1	1	1	1	1	1	1	1	1	1	1	1	1	1	1	1
Transporters	Major facilitator superfamily transporter	PF07690	234	222	234	240	244	244	236	234	235	235	248	247	236	238	244	234	231	232	229	245
	ATP-binding cassette (ABC) transporter membrane domain	PF00664	103	95	96	102	100	98	102	98	97	95	101	99	102	95	107	97	99	96	100	99
	Amino acid permease	PF00324	44	43	41	43	43	44	42	42	42	41	44	44	42	41	43	41	41	45	41	42
	Amino acid permease, type 2	PF13520	24	24	22	23	24	25	23	23	23	22	25	25	23	22	24	22	22	24	22	23
	Oligopeptide transporter	PF03169	22	21	20	20	21	20	21	20	21	21	20	20	21	20	20	20	20	22	20	20
	High-affinity iron permease	PF03239	1	1	1	1	1	1	1	1	1	1	1	1	1	1	2	1	1	1	1	1
	Ammonium transporter	PF00909	2	2	2	2	2	2	2	2	2	2	2	2	2	2	2	2	2	2	4	2
Detoxification / redox	Cytochrome P450 monooxygenase	PF00067	73	70	72	76	73	77	72	70	73	73	78	78	72	70	79	69	73	71	71	77
	Glutathione S-transferase, N-terminal domain	PF02798	23	20	22	20	18	19	22	19	20	19	19	19	22	21	19	21	19	20	22	19
	Glutathione S-transferase, C-terminal domain	PF00043	30	23	26	24	23	25	27	23	24	26	27	26	26	27	25	25	24	26	26	24
	Catalase	PF00199	3	3	3	4	3	3	3	4	4	3	3	3	3	3	3	3	3	4	3	4
	Peroxidase	PF00141	8	7	8	7	9	10	8	7	7	7	10	10	8	8	8	8	8	8	8	8
	Peroxidase, class II	PF01328	2	1	1	0	2	2	1	0	0	0	2	2	1	1	1	1	1	1	0	0
	Copper/zinc superoxide dismutase	PF00080	3	3	3	3	3	3	3	4	3	3	3	3	3	3	3	3	3	3	3	3
	Iron superoxide dismutase	PF00081	3	3	3	3	3	3	3	3	3	2	3	3	3	3	3	3	3	3	3	3
	Thioredoxin	PF00085	15	16	15	15	15	15	15	15	15	14	15	15	15	15	15	15	15	14	15	15
	Glutaredoxin	PF00462	4	4	4	4	4	4	4	4	4	4	4	4	4	4	4	4	4	4	4	4
	Dehydrogenase	PF00106	145	149	146	148	146	152	146	146	145	148	152	152	146	145	147	147	146	145	149	144
	Flavin-containing monooxygenase (non-P450)	PF00743	32	23	24	24	23	27	24	24	22	23	29	28	23	27	26	24	23	25	25	26
Lipases	Lipase	PF03583	16	17	19	19	17	17	21	20	18	18	17	17	19	18	17	18	17	20	18	21
	GDSL-like lipase/acylhydrolase	PF00657	7	6	8	7	6	6	10	8	7	7	6	6	8	7	6	7	6	7	7	9
	α/β-hydrolase fold enzyme	PF00561	79	76	80	84	83	82	79	83	84	79	86	86	79	81	83	81	79	85	81	83
Secreted antimicrobial / interaction	Antifungal protein	PF11402	1	1	1	1	1	1	2	1	1	1	1	1	2	1	1	1	1	1	1	2
	Antifungal peptide	PF11410	1	1	1	1	1	1	1	1	1	1	1	1	1	1	1	1	1	1	1	1
	Aegerolysin family pore-forming protein	PF06355	1	1	1	1	1	1	1	1	1	1	1	1	1	1	1	1	1	1	1	1
	Enterotoxin A–like protein	PF01330	12	13	11	11	12	11	11	12	11	8	10	10	10	9	11	9	9	13	11	16
	Jacalin-like lectin	PF01419	10	8	8	7	8	8	7	6	7	8	8	8	7	8	8	8	8	8	8	7
	Ricin B–like lectin domain	PF00652	6	5	4	4	4	4	4	4	4	4	4	4	4	4	4	4	4	4	4	4
Signal transduction	Protein kinase	PF00069	166	142	153	151	151	155	173	152	148	150	161	160	173	161	163	160	156	156	152	166
	Protein phosphatase 2 C	PF00481	10	9	10	10	11	10	10	10	10	10	10	10	10	10	10	10	10	10	10	10
	Ras small GTPase	PF00071	139	137	139	135	137	139	138	137	137	135	139	138	138	136	138	134	136	136	139	139
	Ras guanine nucleotide exchange factor	PF00617	5	6	6	6	6	6	6	6	6	6	6	6	6	6	6	6	6	6	6	6
	Ras GTPase-activating protein	PF00117	4	4	4	4	4	4	4	4	4	4	4	4	4	4	4	4	4	5	5	4
	G protein–coupled receptor (7-transmembrane receptor)	PF00001	9	9	8	8	8	8	8	8	8	8	8	8	8	8	9	8	8	9	8	8
Transcription factors	Zn₂Cys₆ fungal transcription factor	PF00172	260	257	255	252	257	283	265	261	256	254	281	281	269	273	277	272	254	277	260	273
	Basic leucine zipper transcription factor	PF00170	17	18	17	16	16	17	19	16	16	18	17	17	19	16	17	16	18	18	16	18
	C2H2 zinc finger transcription factor	PF00096	52	52	50	50	51	53	54	53	51	53	53	53	54	59	52	57	53	57	54	56
	Velvet family transcriptional regulator	PF11754	2	3	3	2	2	2	2	2	2	2	2	2	2	2	3	3	3	3	3	2
Stress response	Heat shock protein 70	PF00012	12	12	15	12	13	14	14	12	11	13	14	13	14	17	13	15	16	15	15	16
	Heat shock protein 90	PF00183	1	1	1	1	1	1	1	1	1	1	1	1	1	1	1	1	1	1	1	1

### CAZ enzymes

**Fig. 3:**
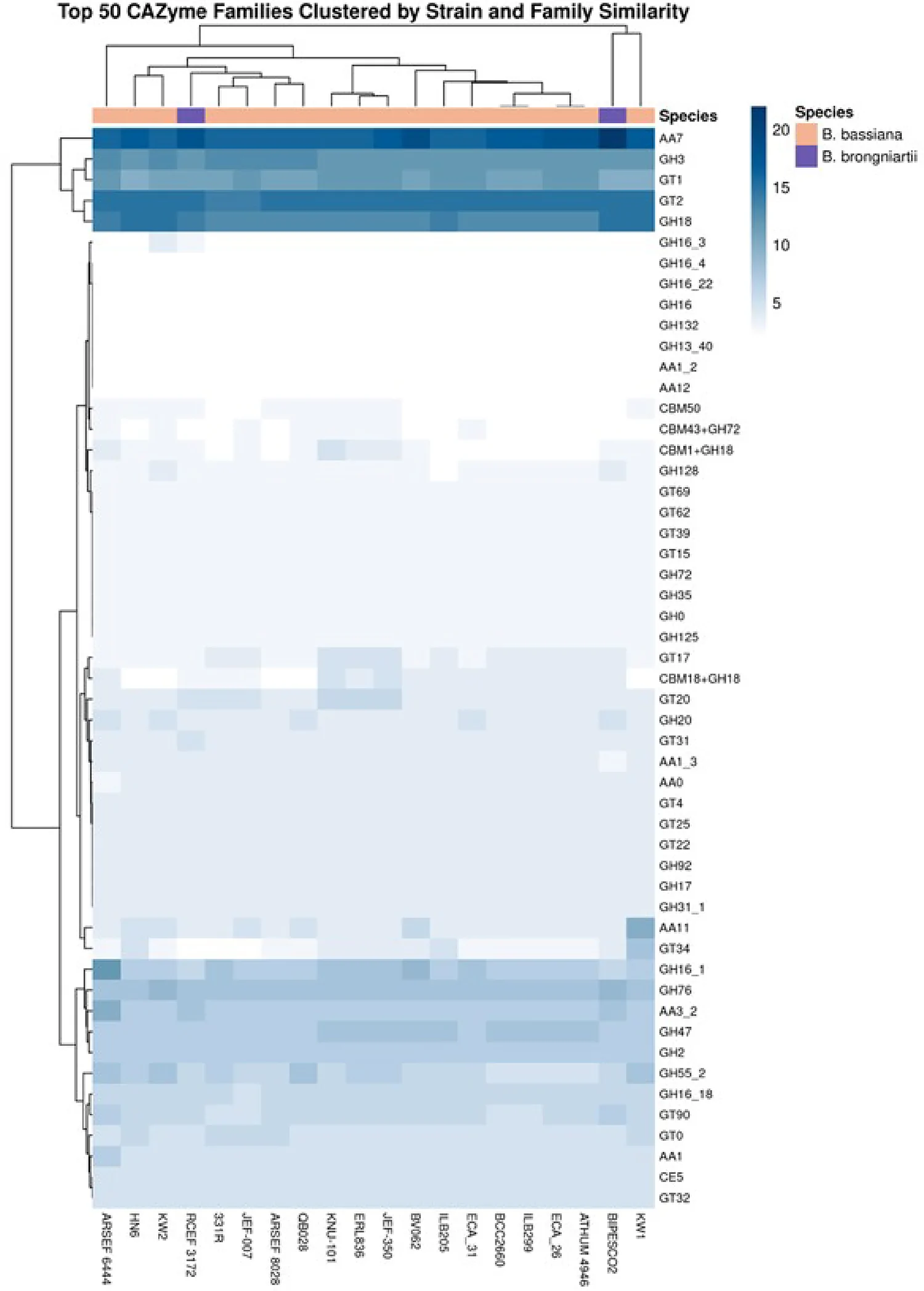
Comparative distribution of the top 50 CAZyme families across 20 *Beauveria* strains. Hierarchical clustering of 50 of the total 150 families based on family abundance, groups strains (columns) and CAZyme families (rows) according to similarity in copy number profiles. The colour scale indicates the number of enzyme copies per family, with darker blue representing higher copy numbers. Strains are annotated by species, with *B. bassiana* shown in magenta and *B. brongniartii* in orange colouring. The heatmap illustrates the strong conservation of CAZyme repertoires across *Beauveria* strains, with only a limited number of families—including GH27 α-galactosidases, GH16_1 glucanases, AA7 oxidases, and PL20 polysaccharide lyases—showing consistent interspecific variation.

**Fig. 4:**
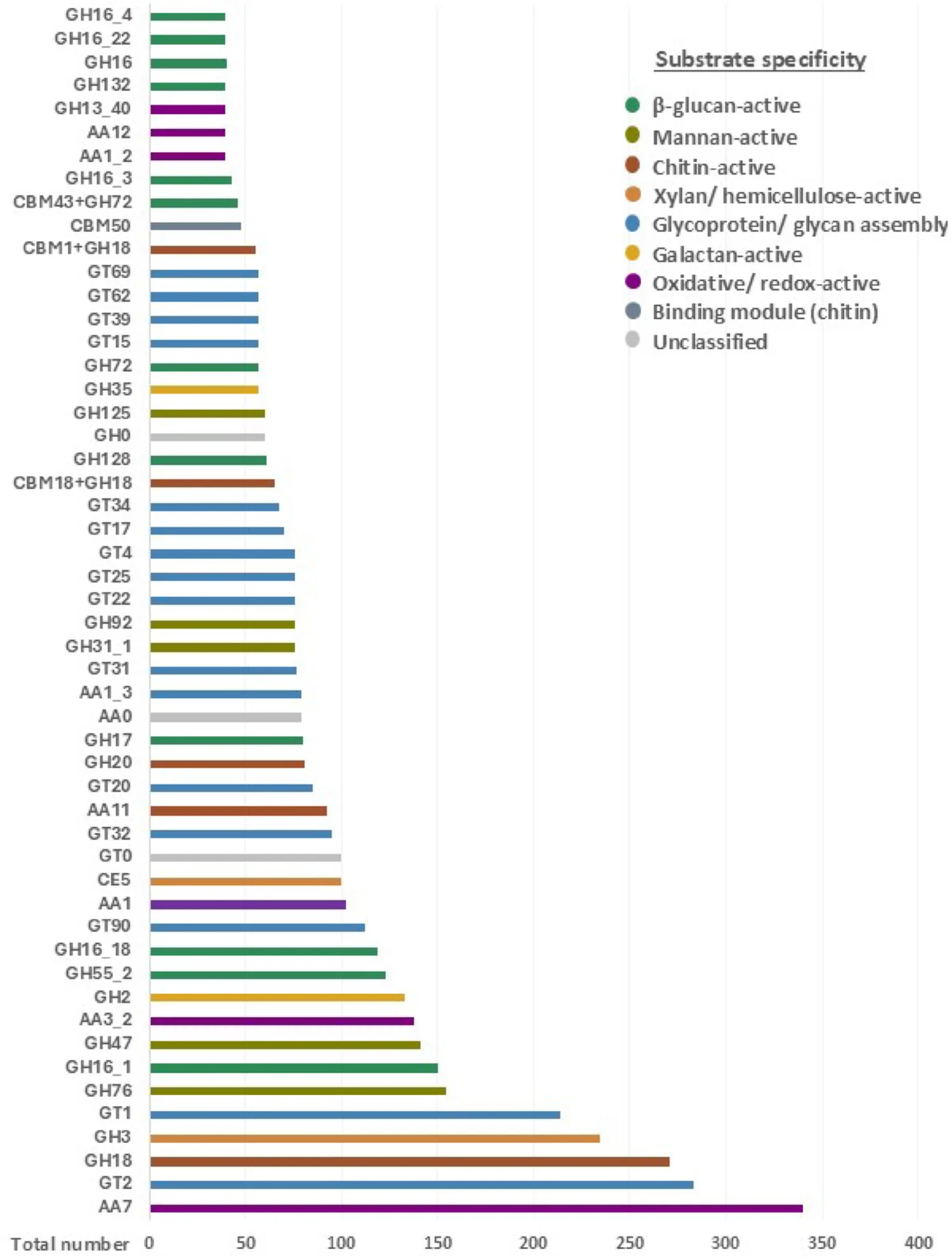
Distribution of the top 50 CAZyme families of *Beauveria* genomes based on primary substrate specificity. The bar plot shows the total number of genes per CAZyme family grouped by predicted substrate specificity: green, β-glucan-active enzymes (GH16_4, GH16_22, GH16, GH132, GH13_40, GH16_3, CBM43 + GH72, CBM50); olive, mannan-active enzymes (GH2, GH35, GH92, GH125, GH47, GH76); brown, chitin-active enzymes (GH18, GH19, CE4/CBM18-GH18, GH20); orange, xylan/hemicellulose-active enzymes (GH10, GH11, GH43, GH62, GH39, CE1–CE5, GT34, GT17, GT4, GT25, GT22); blue, glycoprotein and glycan-assembly GTs (GT0, GT20, GT32, GT15, GT31, GT90); gold, galactan-active enzymes (GH2, GH35, GH53); purple, oxidative/redox-active AA families (AA1, AA3_2, AA7, AA12); gray-blue, chitin-binding modules (CBM18, CBM50); light grey, unclassified or broad-specificity CAZymes.

Carbohydrate-active enzyme (CAZyme) analysis revealed a largely conserved enzymatic repertoire across all *Beauveria* strains, dominated by glycoside hydrolases (GHs) and glycosyltransferases (GTs) (Figs. 3 and 4). A total of 150 different CAZyme families were conserved among all strains, and the number of representatives in each family showed minimal differences (Fig. 3; Table S4). Despite the overall conservation, comparisons uncovered meaningful inter-strain variation, suggesting differential adaptation strategies or functional specialization (Fig. 3; Table S4). Despite the overall conservation of CAZyme repertoires, the most notable patterns of variation involved GH27 α-galactosidases, GH16_1 glucanases, AA7 oxidoreductases, PL20 polysaccharide lyases, and the distribution of multidomain chitin-binding proteins. Both species encoded two GH27 α-galactosidases fused to a CBM13 carbohydrate-binding domain. However, *B. brongniartii* contained an additional GH27 α-galactosidase that lacked an associated CBM13 domain. GH27 enzymes are α-galactosidases that act on terminal α-galactose or N-acetylgalactosamine residues of glycoproteins and galactomannans. The CBM13 domain binds galactose residues, so the fused enzymes are probably optimized for insoluble substrates, while the plain GH27 targets soluble galacto-oligosaccharides, indicating a species-specific diversification of α-galactosidase function.

All *Beauveria* strains encoded a rich repertoire of chitin-active enzymes essential for the degradation of insect cuticle. Among them, the GH18 chitinase family was the most expanded (271 proteins across 20 genomes), with 14 proteins found in each *B. brongniartii* strain and 13 in *B. bassiana* strains. Notably, GH18 enzymes also appear expanded in *Metarhizium* species (Gao et al. 2011). Additional chitin-associated families are the copper-dependent AA11 lytic polysaccharide monooxygenases (LPMOs) and GH20 β-acetylhexosaminidases (Fig. 3). Many of these enzymes were coupled with carbohydrate-binding modules, such as CBM18 (chitin-binding), CBM1 (cellulose/chitin-binding), and CBM50/LysM (peptidoglycan/chitin-binding), that enhance chitin recognition and affinity, contributing to host immune evasion. Notably, a multidomain protein combining CBM18 + CBM50 + GH18 was identified in several isolates — including *B. brongniartii* BIPESCO2 and *B. bassiana* ERL836, JEF-007, JEF350, and KNU-101 — suggesting an expanded chitin-binding and immune-shielding capacity in these strains (Fig. 3; Table S4). Chitin deacetylases (CE4), which convert chitin to chitosan and reduce immune recognition, were also universally present across strains.

Enzymes associated with cell wall remodelling, hyphal growth, and adhesion — including GH16, GH55, GH128, GH132, GH17, and GH72 and composite GH72 + CBM43 families — were among the most abundant CAZymes (Figs. 3 and 4). These β-1,3/β-1,6-glucanases and glucanosyltransferases maintain cell wall integrity, promote hyphal flexibility, and assist in surface adhesion and penetration during infection. Members of the GH16 family, in particular, were highly represented, with 20–25 copies per genome. The virulence-associated subfamily GH16_1 (GH16_fungal_Lam16A_glucanase) was slightly more abundant in *B. bassiana* (7–8 or even 12 copies in some strains) than in *B. brongniartii* (6 copies). This subfamily includes the experimentally characterized host immune evasion glucanase BbEng1 (Wang et al. 2023). The remaining subfamilies (GH16_3, GH16_4, GH16_18, GH16_22, GH16_23) showed no consistent species-specific pattern (Fig. 3, Table S4).

Auxiliary activity (AA) enzymes, which perform oxidative reactions critical for redox homeostasis and virulence, formed another highly expanded group. AA7 (flavin-dependent oxidases) enzymes had considerably more copies in *B. brongniartii* (18–22 compared to 16–17 in *B. bassiana* strains), while AA3_2 (glucose-methanol-choline (GMC) oxidoreductases), and AA1 (multicopper laccases/ferroxidases) were particularly enriched in both species, consistent with roles in redox regulation, reactive oxygen species (ROS) detoxification, and melanin biosynthesis during conidial and hyphal body development. A *B. bassiana* laccase gene has been experimentally found to detoxify insect-generated ROS and interfere with host immune phenoloxidase (PO) activation (Lu et al. 2021). Less represented oxidative families, such as AA5_1 (glyoxal oxidases), AA5_2 (copper radical oxidases), and AA6 (1,4-benzoquinone reductases) are known to contribute to oxidative stress defense, detoxification and extracellular electron transfer. Several glyoxal oxidases (AA5_1) have been characterized in both phytopathogenic and entomopathogenic fungi, contributing to their virulence against plant or insect hosts (Leuthner et al. 2005; Liu et al. 2024). Additionally, benzoquinone reductases (AA6) produced by *B. bassiana* were found to detoxify quinones produced by beetles as antimicrobial compounds, constituting a host-specific virulence factor against the tenebrionid beetle *Tribolium castaneum* (Pedrini et al. 2015). Collectively, these enzymes mediate protection against host-derived ROS and support immune suppression and tissue colonization.

A broad set of glycosyltransferases (GTs) was also detected, spanning GT1, GT2, GT4, GT8, GT15, GT17, GT20, GT22, GT23, GT25, GT31, GT32, GT34, GT39, GT62, GT64, GT69, and GT90 families (Table S4). These enzymes participate in the biosynthesis of chitin, glucans, and complex wall glycans, as well as in protein and secondary-metabolite glycosylation — processes crucial for cell wall assembly, secretion of glycoproteins, and modification of metabolites that enhance adhesion and immune evasion.

Several CAZy families typically associated with plant-associated fungal lifestyles were also present across the *Beauveria* genomes. These included enzymes targeting (primarily) hemicellulose and cellulose (GH5, GH10, GH12, GH43, AA9, and CE1-CE3, CE5) and pectin (PL1, PL3). Interestingly, a polysaccharide lyase family PL20, encoding alginate/uronate lyases, was detected exclusively in *B. brongniartii*. Such enzymes, which cleave uronic-acid-containing polysaccharides via β-elimination, have also been reported in *Fusarium virguliforme* (Chang et al. 2016) and BLAST protein similarity searches also detected similarity in *Metarhizium*, *Pochonia* and *Akanthomyces* species (65–85% identity). Other pectin lyases and cutinases are known to be virulence factors for plant pathogens (Hugouvieux-Cotte-Pattat et al. 1996). The absence of PL20 in *B. bassiana* genomes and its consistent presence in all *B. brongniartii* strains available in the NCBI database were confirmed by BLAST sequence comparison.

Among the most populated families were those acting on mannan- or galactan-rich substrates. While GH2, GH27, GH35, GH92, and GH31 families contribute to the breakdown of plant hemicellulosic side-chains and storage polysaccharides, the highly represented GH47, GH76, and GH125 encode α-mannosidases that primarily remodel fungal cell-wall mannoproteins and process insect N-linked glycoproteins, reflecting conserved roles in fungal development and host immune evasion rather than direct plant colonisation. These proteins were uniformly distributed across all *Beauveria* strains.

### Biosynthetic gene clusters (BGCs)

**Fig. 5:**
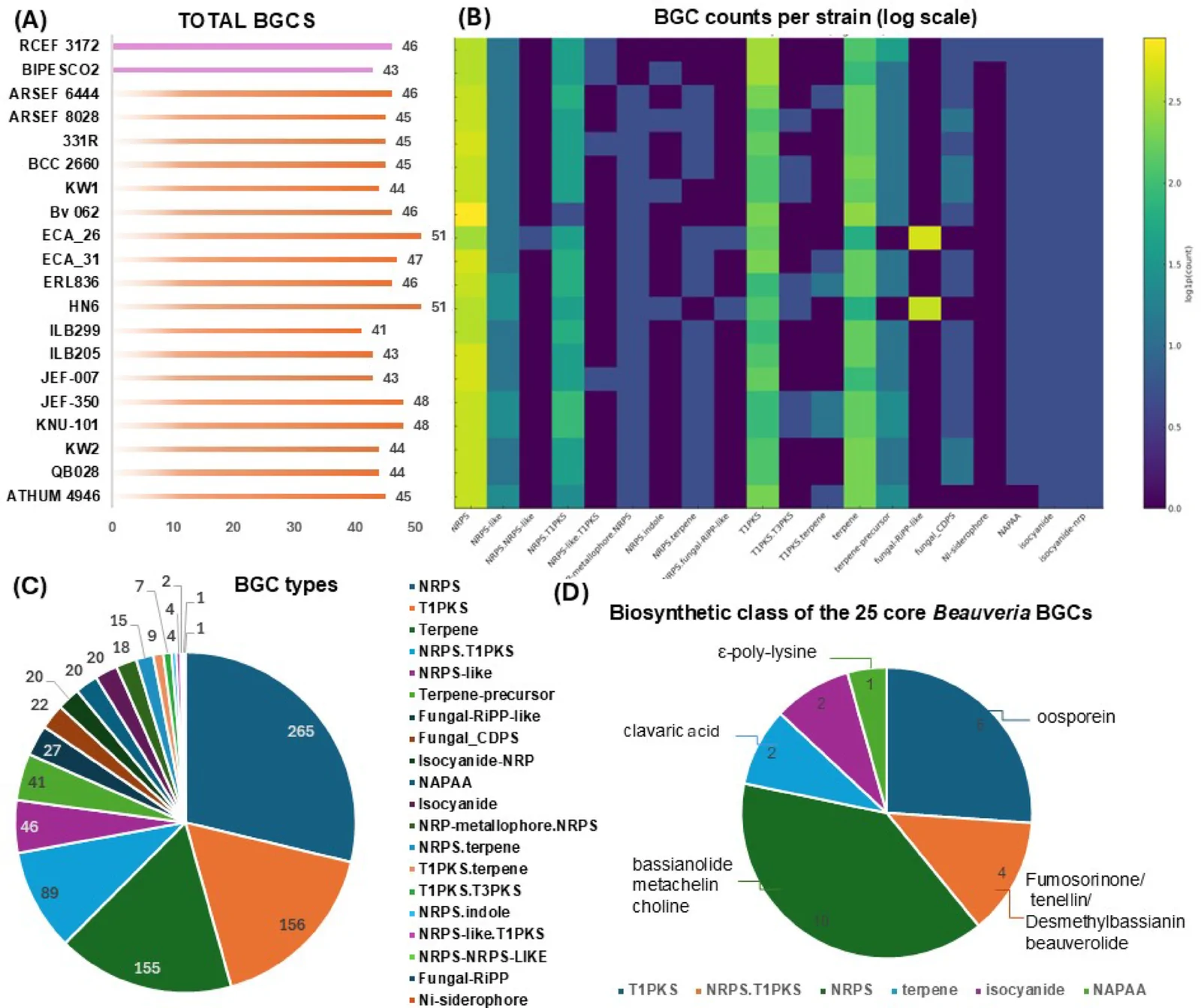
Comparative overview of biosynthetic gene clusters (BGCs) across 20 *Beauveria* genomes. **A** Total number of predicted BGCs identified per strain using antiSMASH. Although BGC counts varied moderately among strains (41–51 clusters), both *B. bassiana* and *B. brongniartii* displayed broadly similar overall BGC loads. **B** Heatmap showing the distribution of BGC classes across strains (log-scaled counts). NRPS, T1PKS, hybrid NRPS-PKS, terpene, and RiPP clusters represent the dominant biosynthetic classes, while siderophores, indoles, and isocyanides occur less frequently. Distinct enrichment patterns are visible across strains, with several species- or strain-specific expansions, particularly within T1PKS-related clusters. **C** Global distribution of the 105 GCF types across the 20 genomes. NRPS clusters constitute the largest proportion of the biosynthetic repertoire, followed by T1PKS and terpene clusters. Hybrid architectures (NRPS-T1PKS, T1PKS-terpene, NRPS-like, and RiPP-like) form a smaller but functionally diverse subset of the pan-genome. **D** Biosynthetic classes of the 25 core BGCs conserved in all *Beauveria* genomes (and according to the MiBIG repository). These include clusters responsible for major entomopathogenic and ecological metabolites such as oosporein, bassianolide, beauverolide, dimethylbassianin, ε-poly-lysine, clavaric acid, metachelin

All 20 *Beauveria* genomes encode on average 46 BGCs each (Fig. 5A). Hierarchical clustering of BGC class composition per strain showed a substantial variation among strains and between species (Fig. 5B). *B. brongniartii* strains have an expanded set of type I polyketide synthase (T1PKS) BGCs (6–9 T1PKS in *B. bassiana* versus 11 in *B. brongniartii*) (Fig. 5B). Cluster network comparative analysis identified a total of 922 BGCs grouped into 105 gene cluster families (GCFs) with various backbone types (Fig. 5C). 28 BGCs were classified as singletons, and they mainly belonged to fungal-RiPP, fungal RiPP-like or NRPS types (Table S5). These 105 GCFs form both common and species-specific families. Out of the 105 GCFs, 25 were present in all 20 *Beauveria* strains, representing the core biosynthetic repertoire of the genus (Fig. 5D). NRPS and NRPS.T1PKS clusters comprised half of these, while only 8 out of the 25 core genus clusters matched the known MiBIG clusters with high or medium similarity, including the insecticidal compounds of oosporein, bassianolide, beauverolide, several siderophores such as ε-poly-lysine, and metachelin, as well as the terpenoids choline and clavaric acid (Fig. 5D). Beauverolides are known to be involved in killing the host during infection, while oosporein is mainly an antimicrobial compound, also promoting infection (de Bekker et al. 2013; Ávila-Hernández et al. 2020; Pedrini 2022). *B. bassiana* is also known to produce bassianin (diketomorpholine), enniatin, and tenellin (2-pyridone) (Eley et al. 2007; Kim et al. 2024). The remaining core *Beauveria* clusters belong to various types, including 1 isocyanide and a hybrid isocyanide-nrp cluster (Fig. 5D). Both are similar to respective clusters in *Metarhizium*, *Akanthomyces*, and other entomopathogens, indicating a potential conserved role in insect virulence.

Network analysis also aided in the identification of 6 species-specific clusters in *B. brongniartii* that were conserved in both strains and absent in all *B. bassiana* strains. These BGCs belong to T1PKS (4), terpene (1), or T1PKS-NRPS-like (1) classes, but none of them matched known compounds (Table S5). The four T1PKS clusters have extensive oxidative tailoring suites (multiple P450s, FMOs, aldo-keto reductases), predicting novel, heavily modified reduced polyketides. One terpene cluster encodes a squalene/sesquiterpene synthase (SQS/PSY), together with several oxidative tailoring enzymes, including an amine oxidase, a short-chain dehydrogenase, a flavin monooxygenase (FMO), and a cytochrome P450. The presence of both an ICMT (Integral Membrane Methyltransferase) methyltransferase and an MFS transporter further supported the production and secretion of an oxygenated terpenoid metabolite. The sixth BGC encodes a hybrid PKS-NRPS system consisting of a PKS module (KS-AT-DH-KR) followed by an NRPS A-T module and multiple tailoring enzymes, suggesting synthesis of a polyketide-peptide hybrid metabolite. The respective BGC of BIPESCO2 matches the compound 6-methylsalicylic acid (BGC0001276) of *Aspergillus terreus*, due to a similarity with the PKS synthase (100% coverage-46% identity), which has been experimentally verified in this fungus. The role of 6-MSA and related compounds is variable (Bejenari et al. 2023). However, the second NRPS synthase domain shows similarity to fungi belonging to Xylariales, like *Hypoxylon fragiforme* and *Apiospora marii* and not with any Hypocrealean fungi, suggesting a horizontal gene transfer event.

Two *B. bassiana*-specific cluster families were present in all 18 *B. bassiana* strains and completely absent in the two *B. brongniartii* strains. The first corresponds to the beauvericin compound, a well-known NRPS implicated in insecticidal activity and virulence, which was not detected by AntiSMASH in *B. brongniartii* strains. However, closer inspection did detect the nucleotide sequence of the cluster, with striking similarity to the *B. bassiana* cluster, but the synthase is truncated (Table S5). The second *B. bassiana*-specific cluster did not match any known MIBiG clusters and the core synthase gene encodes a three-module NRPS with the architecture (C-A-PCP-E/C-A-PCP/C-A-PCP-TD), suggesting the biosynthesis of a cyclic tripeptide containing one D-configured residue and a terminal alanine. The synthase is flanked by multiple tailoring and transport enzymes, including a methyltransferase, a sulfotransferase, an amino oxidase and multiple ABC and MFS transporters, all likely involved in the modification and transfer of the produced compound. Protein BLAST search of the synthase showed results in *Aspergillus mulundensis* (93% coverage and 52% identity), as well as *Trichophyton* sp. and *Arthroderma* sp. (94% coverage and 42% identity), while phylogenetic analysis using the NaPDoS2 database, classified the synthase close to HC-toxin, iturin and fengycylin synthetases, confirming the biosynthesis of a modified cyclic tripeptidic compound.

Besides species-specific clusters, structural divergence of core insecticidal clusters was evident. The hybrid T1PKS-NRPS beauverolide cluster (BGC0002203.2), present in both species, exhibits distinct structural differences in the core biosynthetic NRPS gene that is predicted to alter the chemical outcome of the cluster (Table S5). In both species, the PKS retains a complete reductive module (KS-AT-DH-cMT-ER-KR-ACP), suggesting that the same methyl-branched polyketide starter unit is synthesized. However, in *B. bassiana*, the NRPS protein contains adenylation domains predicted to incorporate an undefined residue (M2), phenylalanine (M3), and D-isoleucine (M4), consistent with the canonical beauverolide cluster. In contrast, *B. brongniartii* lacks the phenylalanine-specific A domain and the terminal D-isoleucine module, instead incorporating two “X” amino acids and a non-aromatic D-X residue (Table S5). Moreover, the synteny is slightly different, since *B. brongniartii* lacks the canonical 2-oxoglutarate-dependent oxygenase found in *B. bassiana*. This Fe(II)-dependent enzyme performs precise hydroxylations that add polar functional groups to the beauverolide scaffold. Instead, all *B. brongniartii* strains possess an N-monooxygenase (NMO) gene at the syntenic locus adjacent to the PKS-NRPS core. The NMO catalyzes flavin- and NADPH-dependent oxidations targeting heteroatoms rather than carbon centers, a shift predicted to yield less hydroxylated, more hydrophobic beauverolide analogues. This consistent substitution in both *B. brongniartii* strains suggests a conserved functional shift in oxidation strategy, likely influencing the ring hydroxylation and polarity of the resulting compounds. AntiSMASH core-scaffold predictions align with these enzymatic differences: *B. bassiana* is predicted to synthesize a canonical beauverolide-like structure containing aromatic and hydroxylated residues, whereas *B. brongniartii* is predicted to produce a more hydrophobic, aliphatic analogue lacking both the aromatic ring and D-Ile hydroxylation. Notably, the FR47-like gene (PF08445), commonly found in beauverolide clusters, is also absent from *B. brongniartii*, but it is unknown whether it contributes to compound production. This coordinated shift in NRPS module composition and oxidative tailoring suggests that the two species generate distinct cyclodepsipeptide profiles despite sharing the same ancestral cluster (Table S5).

Another notable example of divergence is the bassianolide cluster, a cyclic depsipeptide associated with virulence and cytotoxic activity (Xu et al. 2009; Pedrini 2022). Despite the shared NRPS architecture, the two clusters have extensive gene-order rearrangement in tailoring enzymes, indicating that each lineage likely produces distinct structural analogues of the same core scaffold. In *B. brongniartii*, the cluster appears truncated, with several biosynthetic genes missing compared to *B. bassiana*. In *B. bassiana*, the cluster includes two additional oxidoreductases (a cytochrome P450 and an acyl-CoA dehydrogenase), an α/β-hydrolase, and an AMP-dependent ligase, forming an oxidation-rich modification environment consistent with the generation of more hydroxylated or acylated dipeptide derivatives. In contrast, *B. brongniartii* retains only a cytochrome P450 and an α/β-hydrolase, while acyl-CoA dehydrogenase and AMP-dependent ligase are missing. Moreover, it uniquely encodes an aminotransferase. These differences suggest that, although both species likely synthesize the same core dipeptide backbone, the downstream modifications may not be identical. The presence of additional oxidoreductases in *B. bassiana* could enable more oxidative tailoring steps, whereas the aminotransferase unique to *B. brongniartii* may support alternative modifications, but the precise biochemical consequences cannot be inferred solely from gene content. Overall, the conservation of the NRPS core combined with variation in associated tailoring enzymes indicates that the two species may generate related but not necessarily identical compounds, although experimental confirmation would be required to determine the exact structural outcomes.

A total of 328 singleton BGCs were identified across the dataset of both species, indicating strain-specific secondary metabolism and potentially unique bioactivities or ecological functions (Table S5).

### Effector proteins

**Fig. 6:**
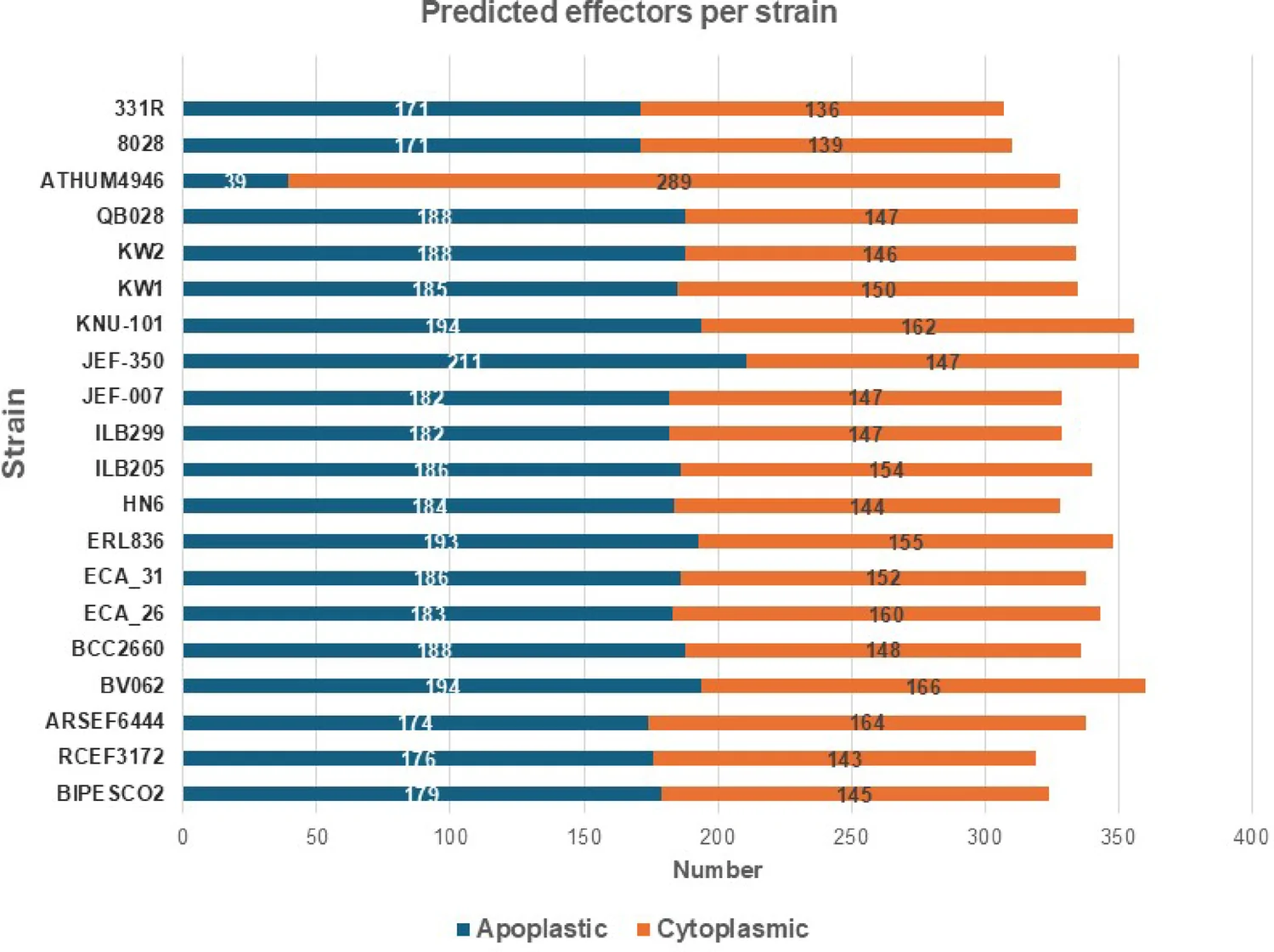
Total predicted cytoplasmic and apoplastic effectors across *Beauveria* strains. Bar plot showing the number of predicted effector proteins per strain, along with the subset predicted to be apoplastic and cytoplasmic. Each bar represents a different *Beauveria* strain, with apoplastic and cytoplasmic effectors highlighted as a subset of the total predicted secreted effector repertoire. Variability in effector content reflects both species-level and strain-specific differences in effector gene composition

Proteins with a predicted N-terminal signal peptide and lacking transmembrane helices (in positions after the first 35 amino acids), were considered as putative effectors and were further analysed with EffectorP (v3.0). Approximately one-third of them were classified as candidate effectors, with an average of 336 effectors per strain (Fig. 6). The majority of the predicted effectors were apoplastic, with no significant differences in apoplastic/cytoplasmic proportions between *B. bassiana* and *B. brongniartii* (Fig. 6). ATHUM 4946 consistently exhibited an atypical apoplastic/cytoplasmic effector distribution following initial analysis as well as reanalysis, which may reflect either a strain-specific biological feature or limitations of computational localization prediction. Similarly to other fungi (Kubicek et al. 2011; Martin et al. 2008), conserved-domain and PHI-base searches (e ≤ 10⁻⁵) in *B. brongniartii* BIPESCO2 strain returned matches for only 50% and 33% of effectors, respectively (Table S6). Interestingly, many of the PHI-base homologs recovered corresponded to proteins previously characterized in phytopathogenic fungi, again suggesting convergence or repurposing of conserved virulence mechanisms. Several apoplastic effectors of *B. brongniartii* BIPESCO2 showed strong similarity to experimentally validated proteins from diverse plant pathogens, including *Exserohilum turcicum* (with a fungal RNase; 58% identity; e = 7 × 10⁻⁴⁶), *Trichoderma virens* (a Cerato-platanin-like protein; 64% identity; e = 2 × 10⁻⁶³), *Colletotrichum gloeosporioides* (a RhoA-like small GTPase; 77% identity; e = 4 × 10⁻¹²³), *Magnaporthe oryzae* MoMAS4 effector (Egh16-like; 57% identity; e = 2 × 10⁻⁹³), a protein implicated in appressorial penetration, as well as to the GH75 chitosanase of *Fusarium solani* (66% identity, e = 4 × 10⁻¹⁴⁰), a negative regulator of pathogenicity through chitosan-mediated host defense signalling (Hadwiger and Beckman 1980; Prapagdee et al. 2007). In addition to these phytopathogenic signatures, several BIPESCO2 proteins also aligned with known virulence-associated proteins from entomopathogenic fungi such as *Beauveria bassiana* and *Metarhizium robertsii* (Table S6), highlighting the mixed ecological ancestry of these effectors.

**Fig. 7:**
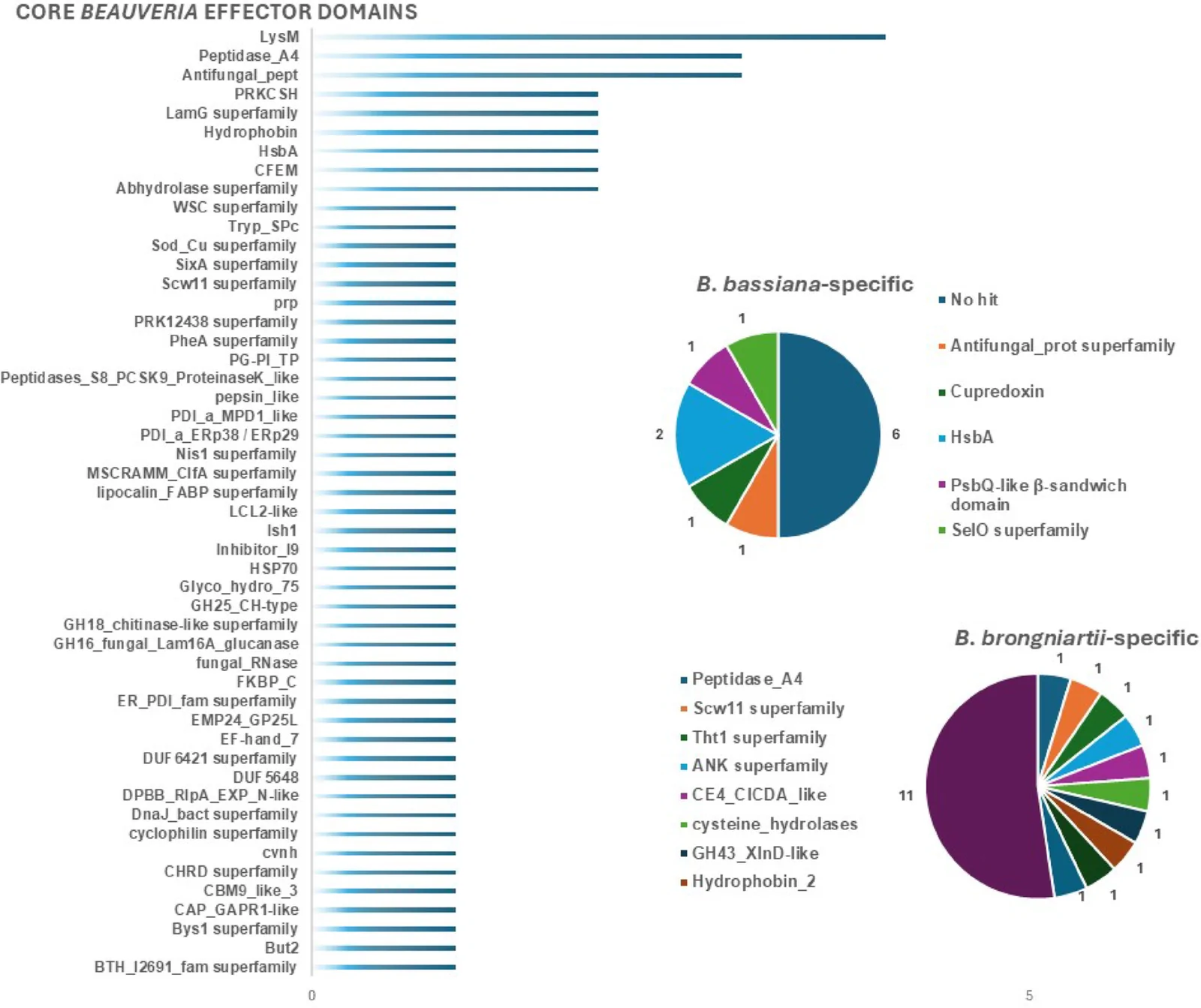
Conserved domain composition of the 130 core *Beauveria* effector orthogroups and species-specific effector orthogroups. Domains were identified using the Conserved Domain Database (CDD). The horizontal bar plot shows the number of core effector orthogroups containing each conserved domain type. Pie charts summarize the conserved domain composition of species-specific effector orthogroups identified in *B. bassiana* and *B. brongniartii*. A large proportion of predicted effectors lacked detectable conserved domains and are indicated as “No hit”

Orthology inference assigned 6,038 out of 6,330 effector proteins (95.4%) to 455 orthogroups, leaving 292 genes (4.6%) unassigned. Half of all assigned genes were contained within the largest 141 orthogroups (O50 = 141), whereas half of all genes in the entire dataset were contained within 149 orthogroups. Across the dataset, a total of 130 *Beauveria* core effector orthogroups were identified (Fig. 7). Within these, 17 were strictly conserved and present in all strains, comprising 10 single-copy orthogroups and 7 with expanded protein families (Table S6). Only 10 out of the 17 orthogroups encoded proteins with conserved domains (e < 10⁻⁵), including proteases (Tryp_SPc), adhesion-related factors that resemble pathogenicity determinants in plant pathogens (CFEM domain) (Kulkarni et al. 2003), and immune-evasion proteins such as the GH16_1 GH16_fungal_Lam16A_glucanase. The remaining seven orthogroups, despite being deeply conserved and in some cases expanded, lacked detectable domains and may represent novel, lineage-specific effector families. The expansion of *Beauveria* core orthogroups to those present in 19 of the 20 genomes (excluding ATHUM 4946, which exhibited unusually low effector numbers) recovered an extended core of 113 additional single-copy effector orthogroups (total to 130). Around half of these (58/113) also lacked identifiable conserved domains. The rest were dominated by LysM domains reflecting conserved roles in chitin binding and immune evasion (Fig. 7; Table S6). Frequent occurrences of proteolytic domains—particularly Peptidase_A4 and Peptidases_S8/ProteinaseK-like—indicate shared strategies for host tissue and cuticle degradation. Moderate representation of hydrolases, including GH18 chitinase-like and GH25 CH-type families, further supports conserved enzymatic roles. Less frequent but notable were adhesion- and stress-related proteins, such as hydrophobins, CAP/GAPR1-like proteins, Hsp70, cyclophilins, and ER_PDI enzymes. Overall, the distribution indicates that the *Beauveria* core effector repertoire is dominated by domain-less SSPs, complemented by a conserved subset of chitin-binding, proteolytic, and stress-response factors. A small number of effector predictions mapped to proteins with well-established intracellular roles (e.g., protein folding, redox homeostasis, or general metabolism). These could be involved in central developmental pathways, in order to control transitions from penetrating germ tubes to hyphal bodies or hyphal bodies to mycelia during the first and later stages of infection. Several such genes have been implicated in virulence mechanisms (Lin et al. 2019). Alternatively, they could be false-positive predictions, retained solely due to secretion signals, and should be interpreted cautiously.

Moreover, we identified 13 *B. bassiana*- and 22 strictly *B. brongniartii*-specific effector orthogroups (Fig. 7; Table S6). Annotated members of *B. bassiana* included antifungal protein-like effectors (cl12933), two HsbA hydrophobic surface-binding proteins—supporting roles in adhesion and host interface interactions—and a cupredoxin fold (cl19115) associated with extracellular redox activity such as protection against host-derived ROS. Additional families included PsbQ-like β-sandwich domains (cl23783) and a SelO adenylyltransferase-like protein (cl19499), both representing conserved but poorly characterized folds in filamentous fungi (Balsera et al. 2003). Similarly, *B. brongniartii*-specific orthogroups included a domain of Peptidase_A4 (cl03371), a hydrophobin_2 (cl6022), a CE4/CICDA-like deacetylase and others (Fig. 7; Table S6). These findings highlight a small but potentially impactful set of species-specific effectors in *B. brongniartii* that may play a direct role in host cuticle degradation and pathogenesis.

## Discussion

This study provides genomic insight into *Beauveria brongniartii*, a comparatively understudied member of the *Beauveria* lineage with a narrow host range. Using long-read sequencing, we generated a high-quality genome assembly of strain BIPESCO2, which currently represents one of the most contiguous assemblies available for the species, with BUSCO completeness and structural integrity comparable to well-annotated *B. bassiana* genomes. This improved reference enabled robust comparative genomic analyses across 20 strains of both species, spanning diverse ecological backgrounds.

Previous genomic studies of *B. bassiana* have revealed a conserved core repertoire of entomopathogenicity-related genes alongside substantial intraspecific diversity (Li et al. 2023; Oberti et al. 2024; Rosana et al. 2021; Solano-González et al. 2023). Our analyses confirm that the two species share a highly conserved core genome consistent with common entomopathogenic strategies. The lack of major differences in core entomopathogenicity-related proteins suggests that specialization in *B. brongniartii* is unlikely to result from large-scale changes in these fundamental gene families. Moreover, unlike what has been observed in *Metarhizium*, where the specialist *M. acridum* encodes fewer proteins than generalist species such as *M. anisopliae* and *M. brunneum* (Gao et al. 2011), we did not detect significant differences in total protein number between *B. bassiana* and *B. brongniartii*. Despite this overall similarity, divergence is clearly evident in specific gene families, secondary metabolism, and effector repertoires, offering insight into how two closely related fungi have adopted contrasting ecological roles. This pattern suggests that differences in host range are unlikely to arise from gains or losses of the core infection machinery itself. Instead, ecological specialization appears to be associated with divergence in specific genomic compartments, including secondary metabolism, lineage-specific orthogroups, and a limited subset of lineage-specific effectors, which may fine-tune host interactions while preserving the ancestral entomopathogenic toolkit.

Orthology analysis revealed strong genomic coherence between species but also identified a distinct set of species-specific orthogroups. *B. brongniartii* encodes several gene clusters absent from all examined *B. bassiana* strains, and tBLASTn validation supported their identification as species-specific features rather than assembly or annotation artifacts. Many of these proteins are associated with immune evasion or cuticle interaction—LysM and GH18 + CBM1 domains, enterotoxin_a, Egh16-like proteins, cuticle-degrading proteases, and CFEM domains linked to iron acquisition. Additional domains related to RNA-binding, ER-mitochondria signalling, and soil-associated toxin systems (e.g., Rhs-like proteins) suggest retention or acquisition of traits favouring soil persistence and interaction with coleopteran larvae. Some domains show the highest similarity to homologues in *Metarhizium* and *Cordyceps*, whereas others resemble bacterial or saprophytic proteins, reflecting a mosaic pattern of lineage-specific retention, possible horizontal gene transfer, or accelerated divergence under ecological selection (Fitzpatrick 2012).

In contrast to *B. brongniartii*, *B. bassiana* shows greater representation in broad-spectrum adhesion factors (HsbA), detoxification enzymes, mucins, and toxin-like proteins. These traits likely support attachment to diverse hosts, survival through the insect gut, and environmental adaptability. The broad functional repertoire retained by *B. bassiana* may also reflect adaptation to ecologically complex environments involving simultaneous interactions with insect hosts, plant-associated niches, microbial competitors, and host-associated microbiota. Recent studies suggest that entomopathogens encounter multifactorial selective pressures within natural systems, including microbiota-mediated resistance and environmental stressors (Zhang et al. 2024; Zhu and Xu 2025), which may favor retention of versatile metabolic and host-interaction capabilities in generalist species. Bacterial-like toxin genes are present in both species, but expansion and variable expression in *B. bassiana* have been reported previously (Xiao et al. 2012). Furthermore, engineered *B. bassiana* strains carrying insecticidal genes from *Bacillus thuringiensis* exhibit enhanced oral toxicity (Qin et al. 2010). Overall, divergence in orthogroup content suggests that *B. brongniartii* is more ecologically restricted, whereas *B. bassiana* has retained or expanded traits associated with ecological breadth. This interpretation is consistent with ecological observations regarding host preference in both species (Rohrlich et al. 2018; Zhang et al. 2025).

In contrast to orthogroups, CAZyme repertoires were nearly identical between species. The core enzymatic machinery for substrate degradation does not appear to be a primary target of divergent evolution within *Beauveria*. These CAZymes are largely conserved, consistent with strong functional constrain, reflecting their essential role across multiple lifestyles (Gao et al. 2011; Xiao et al. 2012). The widespread presence of plant-associated CAZ families—cellulases, hemicellulases, pectinases—is consistent with the documented rhizospheric and endophytic behavior of both species (Lefort et al. 2016; Jaber and Enkerli 2017; Matek et al. 2019; Canassa et al. 2020). This enzymatic toolkit likely represents an ancestral saprophytic repertoire retained for its versatility. Several CAZ families serve dual functions: CE5 cutinases target both plant cuticles and insect epicuticular lipids; AA9 LPMOs cleave crystalline cellulose as well as chitin; GH76, GH43, and GH47 families act on fungal and insect glycoproteins. Such multifunctionality provides a clear evolutionary rationale for their conservation. Although subtle copy-number differences were observed—such as increased AA7 and AA3_2 oxidoreductases in *B. brongniartii* and more GH16_1 enzymes in *B. bassiana*—these differences are modest and unlikely to drive host specialization alone. The strong conservation of CAZyme repertoires despite the ecological divergence between the two species further suggests that host-range specialization in *Beauveria* may not primarily involve large-scale loss of core enzymatic functions. Instead, ecological differentiation may arise through regulatory divergence, including differences in transcriptional activation, promoter architecture, host-induced expression dynamics, or epigenetic regulation (Mierziak and Wojtasik 2024; Armbruster et al. 2026; Gonzalez Ramos et al. 2026).

A contrasting pattern emerges in secondary metabolism. While core insect-associated metabolites such as oosporein, bassianin, beauverolide, and bassianolide are conserved, numerous BGCs show structural remodeling, loss, or expansion. This suggests directional evolution within the secondary metabolite compartment. Notably, *B. brongniartii* encodes nearly more T1PKS clusters as *B. bassiana*. In fungi, T1PKS-derived and hybrid metabolites, such as oosporein, T-toxin, cytochalasins, and aflatoxins function as toxins, oxidative stress modulators, antimicrobial compounds and mediators of various environmental interactions (Brakhage 2013; Keller 2019; Mannino et al. 2021; Wang et al. 2021; Pedrini 2022). In *Beauveria*, PKS synthases have also been linked to development, UV tolerance and cell wall integrity (Meng et al. 2021; Udompaisarn et al. 2020). The unusually rich oxidative tailoring suites identified in several *B. brongniartii-*specific T1PKS clusters suggest an increased potential for diversification of secondary metabolites. This expanded T1PKS repertoire observed in *B. brongniartii* may therefore reflect diversification of chemical interaction strategies associated with persistence in soil environments, adaptation to coleopteran hosts, or modulation of host-derived stress responses, although most predicted clusters remain functionally uncharacterized. In *Metarhizium*, the opposite pattern is observed: generalists encode more NRPS and T1PKS clusters than specialists (Gao et al. 2011). Most predicted BGCs lack characterized products, consistent with previous findings that the majority of secondary metabolite genes in entomopathogenic fungi remain uncharacterized (Pedrini 2022).

Striking divergence is observed in major insecticidal clusters. The bassianolide NRPS cluster in *B. brongniartii* is extensively reorganized. The beauverolide hybrid T1PKS-NRPS cluster shows replacement of the canonical Fe(II)/α-ketoglutarate oxygenase with a flavin-dependent N-monooxygenase and structural differences in the NRPS synthase. The beauvericin NRPS synthase is truncated to the extent that antiSMASH failed to annotate the cluster, and only manual inspection confirmed that most accessory genes remain intact. These changes likely influence metabolite profiles, although functional consequences require experimental validation. Given that beauvericin, bassianolide, benzoquinone, and oosporein contribute to virulence in *B. bassiana* (Xu et al. 2008; 2009; Feng et al. 2015), structural variation may have implications for strain-specific virulence and host range. Consistent with this, Yin et al. (2020) demonstrated production of distinct beauveriolide analogues despite conserved cluster architecture. Preliminary metabolomic observations of our group further suggest variation in bassianolide-like compounds in *B. brongniartii*.

Effector repertoires were largely conserved between species, with similar apoplastic/cytoplasmic distributions and a shared core set across strains. This conserved core includes domains central to host interaction—LysM, hydrophobins, cerato-platanin, and subtilisin-like proteases. Many effectors resemble experimentally characterized proteins from phytopathogens, indicating potential functional conservation beyond insect infection. LysM proteins were consistently present and likely play conserved roles in masking chitin fragments and suppressing host immunity (Bolton et al. 2008; de Jonge et al. 2010; Cen et al. 2017; Zeng et al. 2020). A substantial proportion of predicted effectors lacked recognizable domains, which is common for rapidly evolving secreted proteins under host-driven selection (Todd et al. 2022). Many may therefore represent small, secreted proteins with uncharacterised functions; others may correspond to RNA-based effectors, as small RNAs have been shown to mediate host immune suppression in fungi (Wang et al. 2017; Cui et al. 2019). Together, these findings indicate a conserved but adaptable effector landscape in *Beauveria*. One exception was *B. bassiana* ATHUM4946, which consistently exhibited an atypical effector profile following reanalysis, although whether this reflects genuine strain-specific biology or limitations of computational prediction remains unclear.

Taken together, the data suggest that divergence between *B. bassiana* and *B. brongniartii* is not genome-wide but compartmentalized. Core metabolic capacity and infection machinery remain highly conserved, whereas secondary metabolism and certain lineage-specific gene families exhibit structural and compositional remodeling. The split between the two species is relatively recent (~1.7–2.1 MYA; Shen et al. 2020; Kumar et al. 2022), which likely explains the strong conservation of CAZymes and core orthologues. This conserved toolkit supports saprotrophy, rhizosphere competence, and fundamental stages of entomopathogenic infection, as seen in other Hypocrealean EPF (Xiao et al. 2012; Hu et al. 2014; Wang et al. 2023). It likely reflects a broad ancestral lifestyle rather than narrow host specificity. In natural environments, such ancestral ecological versatility may involve adaptation not only to diverse hosts but also to complex multitrophic interactions involving plants, microbial competitors, and host-associated microbiota (Zhang et al. 2024; Zhu and Xu 2025). Together, these observations support the view that the contrasting ecological strategies of the two species have evolved primarily through remodeling of specialized genomic compartments rather than through large-scale changes to the conserved core genome.

By contrast, secondary metabolism appears to be a principal axis of divergence. Structural remodeling of BGCs, variation in PKS cluster composition, and modification of canonical insecticidal clusters suggest that chemical ecology may have played a central role in niche differentiation. Importantly, specialization in *B. brongniartii* does not coincide with genome reduction. Instead, the pattern is more consistent with partial remodeling of broad-spectrum virulence chemistry combined with diversification of other metabolite systems. This resembles patterns described in *Metarhizium*, where host specificity aligns more strongly with toxin gain or loss than with CAZyme content (Wang et al. 2012; Hu et al. 2014).

Specialist *Metarhizium* species often show accelerated evolution alongside smaller gene repertoires (Brancini et al. 2018; Moonjely and Bidochka 2019). Our data only partially match this model. While *B. brongniartii* exhibits divergence in BGCs and virulence-associated loci and forms numerous species-specific orthogroups, total gene number remains comparable to *B. bassiana*. This suggests that ecological specialization within *Beauveria* may not follow a simple trajectory of progressive reduction. Instead, specialization may involve targeted remodeling of interaction-related genomic compartments while retaining a broadly competent ancestral framework.

Ultimately, determining whether *B. brongniartii* represents a highly specialized lineage or a lineage with retained ecological flexibility will require broader sampling across the genus and functional validation linking genotype to phenotype. Nevertheless, ecological observations of long-term co-occurrence between European *B. brongniartii* strains and *Melolontha* populations, including partial host resistance to native isolates (Keller et al. 1999; Enkerli et al. 2004; Pedrazzini et al. 2024), are consistent with prolonged host-pathogen interaction in soil environments. This ecological context aligns with the genomic signal observed here: strong conservation of the core infection toolkit, but pronounced divergence in secondary metabolism and niche-associated loci.

## Supplementary Information

Below is the link to the electronic supplementary material.

Supplementary Material 1

Supplementary Material 2

Supplementary Material 3

Supplementary Material 4

Supplementary Material 5

Supplementary Material 6

## Data Availability

The original contributions presented in this study are included in the article. Further inquiries can be directed to the corresponding author(s).
